# Investigating the relationship between carbapenemase production and biofilm formation in *Klebsiella pneumoniae* clinical isolates

**DOI:** 10.1186/s13104-024-06708-9

**Published:** 2024-02-15

**Authors:** Nora M. El Naggar, Riham M. Shawky, Fathy M. E. Serry, Mohamed Emara

**Affiliations:** 1 Faculty of Pharmacy, Department of Microbiology and Immunology, Helwan University, POX 11795, Ain Helwan, Cairo, Egypt; 2 Faculty of Pharmacy, Department of Pharmaceutical Microbiology, Zagazig University, Zagazig, Egypt

**Keywords:** *Klebsiella pneumoniae*, Carbapenem resistance, NDM-1, OXA-48, Biofilm

## Abstract

**Objective:**

Carbapenemase production and biofilm formation in *K. pneumoniae* are crucial factors influencing the pathogenicity and antibiotic resistance of this bacterium. This study investigated the interplay between carbapenemase production and biofilm formation in *K. pneumoniae* clinical isolates.

**Results:**

The distribution of biofilm-forming ability significantly differed between carbapenemase-producing (CP-Kp) (*n* = 52) isolates and carbapenemase-nonproducing (CN-Kp) isolates (*n* = 37), suggesting a potential link between carbapenemase production and biofilm formation. All the *bla*_NDM-1_-harbouring isolates demonstrated biofilm formation, with varying levels classified as strong (33.33%), moderate (22.22%), or weak (44.45%). *bla*_NDM-1_ and *bla*_KPC_-coharbouring isolates did not exhibit strong or moderate biofilm formation. *bla*_NDM-1_ and *bla*_OXA-48_-coharbouring isolates were predominantly moderate (48.65%), followed by weak (32.43%), with none showing strong biofilm production. These findings suggest a correlation between the presence of carbapenemases and biofilm-forming ability; however, the heterogeneity in biofilm-forming abilities associated with different carbapenemase types and the absence of strong biofilm producers in the detected carbapenemase combinations prompt a closer look at the complex regulatory mechanisms governing biofilm formation in CP-Kp isolates.

**Supplementary Information:**

The online version contains supplementary material available at 10.1186/s13104-024-06708-9.

## Introduction

*K. pneumoniae* is a clinically significant gram-negative pathogen known to cause various infections in hospital and community settings [1]. *K. pneumoniae* develops carbapenem resistance, mainly through the production of carbapenemases along with other mechanisms, such as outer membrane impermeability and efflux pumps [2]. The most clinically significant carbapenemases in *K. pneumoniae* are classified into Ambler class A β-lactamases (encoded by *bla*_KPC_), class B metallo-β-lactamases (MBLs) (encoded by *bla*_NDM−1_ and *bla*_VIM_), and class D β-lactamases (encoded by *bla*_OXA−48_) [3, 4]. The emergence and spread of carbapenemase-producing *K. pneumoniae* (CP-Kp) strains have posed a serious threat to public health and continue to occur at alarming rates [5] due to their extensive antibiotic resistance [6], including resistance to carbapenem antibiotics, which are considered the last resort for the treatment of infections caused by multidrug-resistant (MDR) *K. pneumoniae* [7].

*K. pneumoniae* has the ability to produce virulence factors that play a role in its pathogenesis, including the crucial virulence trait of biofilm formation. The biofilm is a complex matrix that frequently consists of a dense matrix of proteins, polysaccharides, and DNA [8, 9], within which bacteria are highly resistant to antibiotics and host immune responses [10], making it challenging to eradicate. Moreover, biofilms of *K. pneumoniae* that develop on medical devices such as catheters and endotracheal tubes pose a substantial risk of infection for patients who are catheterized [11].

The development of antibiotic resistance is often intertwined with infection, highlighting its close association with virulence. This connection becomes especially apparent in the cases of microorganisms capable of producing biofilms [12]. Consequently, both the formation of biofilms and the production of carbapenemases contribute to heightened levels of antibiotic resistance. The aim of this study was to investigate the correlation between carbapenemase production and biofilm formation in *K. pneumoniae* clinical isolates.

## Methods

### Bacterial strains

Eighty-nine *K. pneumoniae* clinical isolates were included in this study (52 CP-Kp and 37 CN-Kp) from different clinical sources: urine (*n* = 38), blood (*n* = 19), wound (*n* = 9), sputum (*n* = 6), pus (*n* = 5) and other (*n* = 12), collected between March 2021 and January 2022 from Egypt Air Hospital in Cairo, Egypt. Identification of the isolates was carried out using matrix-assisted laser desorption/ionization time-of-flight mass spectrometry (MALDITOF/MS, SAI, UK) with score values ranging from 0.82 to 0.87, as per the manufacturer’s recommendations.

### Antimicrobial susceptibility testing

Antimicrobial susceptibility testing was employed by the disk diffusion technique using Mueller-Hinton agar (Oxoid, Thermo Fisher Scientific), following the guidelines established by the Clinical and Laboratory Standards Institute (CLSI) [13]. Imipenem (10 µg), meropenem (10 µg), and ertapenem (10 µg) (Oxoid, Thermo Fisher Scientific) were tested. *E. coli* ATCC 25922 was used as the control strain. Interpretation of zones of inhibition was performed according to CLSI guidelines [14]. The inclusion criterion for the CN-Kp group was based on susceptibility, which was defined as having phenotypes sensitive to all tested carbapenems.

### Phenotypic detection of carbapenemases

Carbapenemase-positive isolates were detected using the modified carbapenem inactivation method (mCIM) in accordance with the CLSI guidelines [14].

### DNA extraction

To obtain crude DNA, 2 colonies were lysed in 500 µL of sterile distilled water at 100 °C for 10 min, followed by centrifugation. The supernatant was stored at − 80 °C for subsequent PCR assays. The concentration and purity of the DNA extract were detected using a NanoDrop spectrophotometer at wavelengths of 260 and 280 nm.

### Detection of resistance genes by PCR

The carbapenemase-encoding genes *bla*_IMP_, *bla*_VIM_, *bla*_KPC_, *bla*_NDM-1_, *bla*_SPM_, and *bla*_*OXA-48*_ were amplified as follows: One multiplex PCR for the detection of *bla*_KPC_, *bla*_NDM-1_, and *bla*_OXA-48_ and three uniplex PCRs for the detection of *bla*_IMP_, *bla*_VIM_, and *bla*_SPM_ were carried out in a 25 µL volume using *COSMO PCR RED* master mix (Willowfort, Birmingham, England). Primers (*Invitrogen*^*®*^, Thermo Fisher Scientific Inc., MA, USA) sequences and sizes are listed in Table 1, and the PCR conditions were as follows: For the multiplex PCR, 10 min at 94 °C and 30 cycles of amplification consisting of 30 s at 94 °C, 40 s at 52 °C, and 50 s at 72 °C were used, with 5 min at 72 °C for the final extension. For the three uniplex PCRs, 10 min at 94 °C and 30 cycles of amplification consisting of 30 s at 94 °C, 40 s at 55 °C, and 50 s at 72 °C, with 5 min at 72 °C for the final extension. A control without a template was included. The amplicons were separated by electrophoresis on a 2% (w/v) agarose gel containing ethidium bromide (0.5 µg/ml) using *Thermo Scientific™ GeneRuler™* 100 bp DNA Ladder (Thermo Fisher Scientific Baltics UAB, Lithuania). Prior to their use in the multiplex PCR assay, the primer pairs were individually tested to confirm their functionality and specificity.

**Table 1:** Primers used for detection of carbapenemase genes

Gene	Primer DNA sequence	Amplicon size (bp)	Reference
*bla*_NDM−1_	F-5’GGTTTGGCGATCTGGTTTTC 3’ R-5’CGGAATGGCTCATCACGATC 3’	621 bp	[15]
*bla*_KPC_	F-5’CGTCTAGTTCTGCTGTCTTG 3’ R-5’CTTGTCATCCTTGTTAGGCG3’	798 bp	[15]
*bla*_OXA−48_	F-5’GCGTGGTTAAGGATGAACA3’ R-5’CATCAAGTTCAACCCAACCG3’	438 bp	[15]
*bla*_IMP_	F-5’GGAATAGAGTGGCTTAAYTCTC3’ R-5’GGTTTAAYAAAACAACCACC3’	232 bp	[16]
*bla*_SPM_	F-5’AAAATCTGGGTACGCAAACG3’ R-5’ACATTATCCGCTGGAACAGG3’	271 bp	[16]
*bla*_VIM_	F-5’GATGGTGTTTGGTCGCATA3’ R-5’CGAATGCGCAGCACCAG3’	390 bp	[16]

### Biofilm assay

The microtiter plate (96-well plate) assay was used to study biofilm formation, as described elsewhere [17]. The bacterial isolates were grown overnight at 37 ℃, and the bacterial suspension turbidity was adjusted to match the OD of a 0.5 McFarland standard in saline using a spectrophotometer (*GeneQuant*, Biochrom Ltd., England). Twenty µL aliquots of each suspension were added to the wells of polystyrene microtiter plates containing 180 µL TSB supplemented with 1% glucose, with three wells per bacterial isolate. The plate was incubated for 48 h under static conditions, and the broth was gently aspirated. Each well was washed thrice with 300 µL of PBS at pH 7.2, and the adherent biofilm layer in each well was stained with 150 µL of 0.5% (w/v) crystal violet solution for 14 min at room temperature. Crystal violet was removed using sterile distilled water, and the plates were air-dried. The biofilm was solubilized by adding 150 µL of 95% ethanol. The OD of each well was measured at 570 nm using a microtiter plate reader (Synergy 2, BioTek, WI, USA). Sterile broth was used as a negative control. Each assay was performed in triplicate on three occasions. The results were interpreted according to the following criteria: no biofilm production (OD_sample_<OD_control_), weak biofilm production (OD_control_<OD_sample_<2xOD_control_), moderate biofilm production (2xOD_control_ < OD_sample_<4xOD_control_), and strong biofilm production (4xOD_control_ < OD_sample_) [17].

### Data analysis

Chi-square tests (Fisher’s exact test where appropriate) were performed using GraphPad Prism version 5.01 for Windows and GraphPad InStat version 3.05 (GraphPad Software, San Diego, California, USA) to assess the relationship between carbapenemase production and biofilm formation. In this context, any correlation analyses that resulted in *p* values less than 0.05 were statistically significant.

## Results

### Characterization of isolates

Among the CP-Kp isolates, 37 exhibited simultaneous resistance to all three tested carbapenems. Specifically, all CP-Kp isolates were resistant to ertapenem, with the exception of two isolates that showed intermediate resistance. Out of 52 CP-Kp isolates, 46 were resistant to meropenem and 37 to imipenem, with intermediate resistance in four and five isolates, respectively. In contrast, 10 isolates were found to be sensitive to imipenem, and two isolates were sensitive to meropenem.

### Prevalence and distribution of carbapenemase genes

**Fig. 1:**
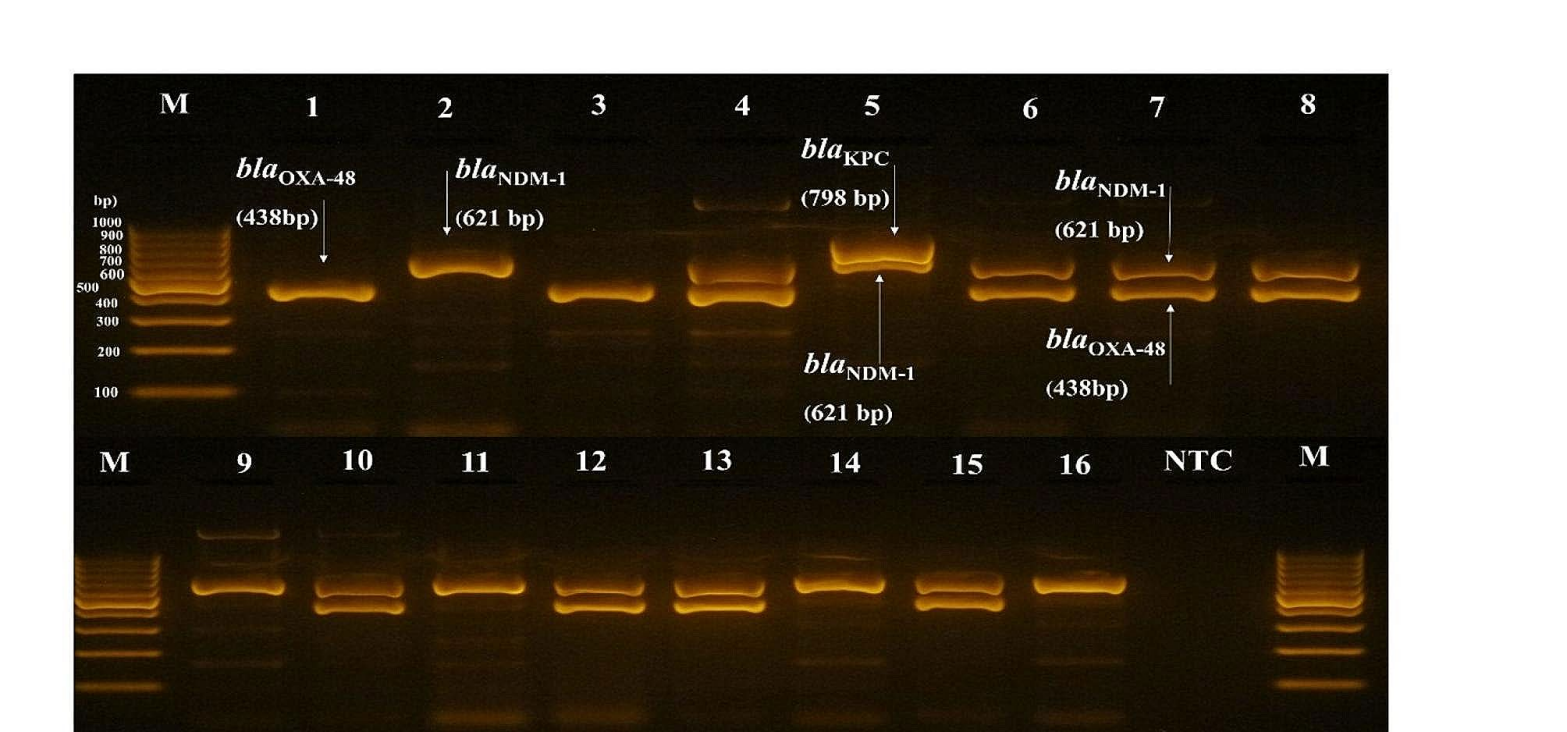
Carbapenemase gene amplification profile in *K. pneumoniae* using multiplex PCR. The PCR products were separated on a 2% agarose gel. A molecular size marker (in bp, measurements on the left, middle, and right) was used, featuring a reference band at 500 bp. No template control (NTC) was included. The specific lanes correspond to the following amplified genes: Lane 1 and 3 for *bla*_OXA-48_ (438 bp); Lane 2, 9, 11, 14 and 16 for *bla*_NDM-1_ (621 bp); Lane 5 for *bla*_NDM-1_ and *bla*_KPC_ (621 and 798 bp); and Lane 4, 6, 7, 8, 10, 12, 13 and 15 for *bla*_NDM-1_ and *bla*_OXA-48_ (621 bp and 438 bp)

All CR-Kp isolates that phenotypically expressed carbapenemase, as determined by the mCIM, were positive for one or more carbapenemase genes, according to PCR results (Fig. 1). Out of the 52 CP-Kp isolates, the *bla*_NDM-1_ gene was detected in 49 (94.23%), and *bla*_OXA-48_ was detected in 40 (76.92%) isolates. None of the carbapenemase genes tested (*bla*_VIM,_*bla*_IMP_, and *bla*_SPM_) were detected. Out of the 52 carbapenemase gene-carrying isolates, 37 (71.15%) coharboured *bla*_NDM-1_ and *bla*_OXA-48_, while 3 (5.77%) isolates carried both *bla*_NDM-1_ and *bla*_KPC_. None of the isolates were found to harbour *bla*_KPC_ only.

### Evaluation of biofilm formation

In total, 63 out of 89 (70.79%) isolates were identified as biofilm producers, and 26 isolates (29.21%) were biofilm non-producers from different clinical sources. The distribution of biofilm-forming ability varied significantly (*p* = 0.0037; chi-square test) between the CP-Kp and CN-Kp isolates. For the CP-Kp isolates, 42.31% exhibited moderate biofilm production, 34.62% were weak producers, 5.77% were strong producers, and 17.31% were non-producers. Among the CN-Kp isolates, 37.84% were weak producers, 10.81% were moderate producers, 5.41% were strong producers, and 45.95% were non-producers (see Table 2 and Supplementary Fig. 1). The biofilm-forming abilities of *K. pneumoniae* isolates were assessed based on various carbapenemase types. Among the *bla*_NDM-1_-harbouring isolates, 33.33% exhibited strong biofilm formation, 22.22% had moderate biofilm formation, and 44.45% had weak biofilm formation. None of the isolates were classified as biofilm non-producers. In *bla*_NDM-1_ and *bla*_KPC_-coharbouring isolates, 66.67% were weak biofilm producers, and 33.33% were biofilm non-producers. In *bla*_NDM-1_ and *bla*_OXA-48_-coharbouring isolates, no strong biofilm producers were observed. Instead, 48.65% were moderate producers, 32.43% were weak producers, and 18.92% were non-biofilm producers. In the *bla*_OXA-48_ harbouring isolates, 66.67% were classified as moderate biofilm producers, and 33.33% were biofilm non-producers. (see Supplementary Table 1).

**Table 2:** Comparison of biofilm-forming ability between carbapenemase-producing (CP-Kp) and nonproducing *Klebsiella pneumoniae* (CN-Kp) isolates (Fisher’s exact test)

Biofilm formation	CP-Kp, *n* = 52 (%)	CN-Kp, *n* = 37 (%)	Total, *n* = 89 (%)	*p* value
No biofilm	9 (17.31)	17 (45.95)	26 (29.21)	0.0046
Weak	18 (34.62)	14 (37.84)	32 (35.96)	0.8243
Moderate	22 (42.31)	4 (10.81)	26 (29.21)	0.0018
Strong	3 (5.77)	2 (5.41)	5 (5.62)	1.0000

## Discussion

Over the past few years, there has been a notable increase in the occurrence of CP-Kp in hospitals worldwide. In this study, 52 CP-Kp isolates were included. *bla*_NDM-1_ was the most predominant (94.23%) carbapenemase-encoding gene detected, followed by *bla*_OXA-48_ (76.92%). This finding is in line with previously reported data that found that 35 out of 37 (94.59%) of their carbapenemase-producing isolates harboured *bla*_NDM-1_ and 26 (70.27%) harboured *bla*_OXA-48_ [18]. In contrast to our findings, another study reported that *bla*_OXA-48_ (25/62, 40.32%) was the most predominant and that *bla*_NDM-1_ had only a minor incidence (6/62, 9.68%) [19]. When a carbapenemase-producing isolate carries multiple carbapenemases, it becomes highly resistant to treatment as it increases the range of hydrolytic activity, making it challenging to target with antibiotics [20]. Here, we found that the coexistence of *bla*_NDM-1_ and *bla*_OXA-48_ accounted for 71.15% of the CP-Kp isolates. This high occurrence is consistent with previous work, which reported a prevalence of 64.86% (24 out of 37 isolates) of tested CP-Kp isolates [18]. In contrast, a previous study conducted in the ICUs of Zagazig University Hospitals reported that only 5.71% (6 out of 105 isolates) of tested CP-Kp isolates coharboured *bla*_NDM-1_ and *bla*_OXA-48_ [21]. In this study, we found a low incidence (5.77%) of CP-Kp isolates that coharbored *bla*_NDM-1_ and *bla*_KPC_. These findings are in accordance with previous studies conducted in Egypt, which reported a low incidence (11.29%) (7 out of 62 isolates) [19] or absence [18] of the *bla*_KPC_ gene in their CP-Kp isolates. This suggests that KPC is less prevalent in our geographic area. However, opposite findings were reported by others who found a higher prevalence of *bla*_KPC_ at 17.14% (18 out of 105) in the tested CP-Kp [21]. Here, we could not detect the presence of *bla*_IMP_ or *bla*_VIM_ in any of our CP-Kp isolates. These findings are in line with other studies conducted in Egypt and other countries that reported the absence of these genes in their CP-Kp isolates [18, 21]. However, in contrast to our findings, some studies reported a higher prevalence [19]. Here, we could not detect the presence of *bla*_SPM_ in any isolate.

One of the important virulence factors of *K. pneumoniae* is its ability to form biofilms [22]. The current study revealed that the majority of the *K. pneumoniae* isolates were biofilm producers (70.79%), regardless of their clinical source. This finding is consistent with that of a study conducted in 2023 [23]. However, a previous study reported a greater propensity for biofilm formation, with 91.2% of the isolates forming biofilms [24]. In this study, there was a significant difference in biofilm-forming ability between CP-Kp and CN-Kp (*p* = 0.0037; chi-square test). These findings corroborate those of the 2022 study on reference strains of *K. pneumoniae* [25]. Notably, a smaller percentage of CP-Kp isolates (17.31%) were biofilm non-producers compared to CN-Kp isolates (45.95%) (*p* = 0.0046; Fisher’s exact test). Therefore, the presence of carbapenem resistance, indicated by the presence of carbapenemases, suggested a greater overall propensity for biofilm formation in *K. pneumoniae*. This could be attributed to several factors, such as the interplay of carbapenemases with regulatory pathways governing biofilm formation, which may lead to the upregulation of key biofilm-associated genes in CP-Kp isolates [25]. Additionally, the environmental stress induced by the presence of carbapenemases might support the formation of biofilms as a survival strategy in CP-Kp isolates. Despite the suggested association, it is noteworthy that there is a subset of CP-Kp isolates that demonstrated an inability to produce biofilms, suggesting that carbapenem resistance in *K. pneumoniae* alone does not guarantee biofilm formation and is likely multifactorial.

CP-Kp isolates, despite being primarily biofilm producers, display varied biofilm-forming capabilities. The strong biofilm producers of the CP-Kp group were exclusively found among the isolates harbouring only *bla*_NDM-1_, consistent with the findings of a previous study [25]. Notably, the *bla*_NDM-1_ and *bla*_KPC_-coharbouring isolates did not exhibit strong or moderate biofilm formation, and one such isolate was a biofilm non-producer. This finding aligns with that of a prior study [23]. In contrast, the majority of *bla*_NDM-1_ and *bla*_OXA-48_coharbouring isolates exhibited a moderate biofilm phenotype (48.65%), followed by a weak biofilm phenotype (32.43%), while 18.92% of the isolates exhibited no biofilm formation. No strong biofilm producers were identified.

Most of the literature indicated a higher prevalence of strong biofilm producers among β-lactamase producers [26, 27]. In the present study, unexpectedly, no correlation was observed between the formation of strong biofilms and the presence of detected carbapenemases. Additionally, none of the isolates detected with more than one resistance gene exhibited strong biofilm formation, contrary to the findings of a recent study [23]. Conversely, another study revealed a notable association between the prevalence of the *bla*_VIM1_ and *bla*_IMP1_ genes and the formation of strong biofilms [24]. These findings suggest that certain carbapenemase subtypes or combinations may enhance biofilm formation, while others may have a more modest impact or even a negative influence. However, other factors, such as quorum sensing [25], the HMV phenotype [28], high adhesion capacity, and cell death, have been reported to have significant impacts on the formation of strong biofilms on *K. pneumoniae* isolates [29].

In conclusion, this study suggested a link between carbapenemases and biofilm formation, but the diverse biofilm-forming capabilities of various carbapenemase types and the lack of strong producers in the detected combinations emphasise the need for a deeper understanding of the intricate regulatory mechanisms governing biofilm formation in CP-Kp isolates.

### Limitations

One of the limitations of this study is the lack of further carbapenemase subtyping and correlation with biofilm phenotypes, which could provide insights into the varying effects of specific carbapenemases or combinations on biofilm formation. In addition, performing functional assays, such as gene knockout experiments or overexpression studies, can validate the role of specific genetic elements in biofilm formation.

### Electronic supplementary material

Below is the link to the electronic supplementary material.

Supplementary Material 1: **Supplementary Figure 1.** Distribution of biofilm-forming ability among carbapenemase-producing (CP-Kp) and nonproducing Klebsiella pneumoniae (CN-Kp) isolates. **Supplementary Table 1.**. Distribution of the biofilm-forming ability across different carbapenemase types in carbapenemase-producing Klebsiella pneumoniae (CP-Kp) isolates

## Data Availability

All data generated or analysed during this study are included in this article and its supplementary information files.

## Abbreviations

ATCC: American Type Culture Collection
*bla*: Beta-lactamase genes
CP-Kp: Carbapenemase-producing *Klebsiella pneumoniae*
CN-Kp: Carbapenemase-nonproducing *Klebsiella pneumoniae*
CR-Kp: Carbapenem-resistant *Klebsiella pneumoniae*
CLSI: Clinical and Laboratory Standards Institute
dNTP: Deoxynucleoside triphosphate
DNA: Deoxyribonucleic acid
eDNA: Extracellular DNA
EPS: Exopolysaccharides
E. coli: Escherichia coli
HMV phenotype: Hypermucoviscous phenotype
ICU: Intensive Care Unit
IMP: Imipenemase
*K. pneumoniae*: *Klebsiella pneumoniae*
KPC: *Klebsiella pneumoniae* carbapenemase
MALDITOF/MS: Matrix-Assisted Laser Desorption-Ionization-Time of Flight Mass Spectrometry
mCIM: Modified Carbapenem Inactivation Method
MDR: Multidrug-resistant
MBLs: Metallo-β-lactamases
NDM-1: New Delhi metallo-β-lactamase type 1
OD: Optical density
OXA-48: Oxacillinase-48
PBS: Phosphate Buffered Saline
PCR: Polymerase Chain Reaction
SPM: Sao Paulo metallo-β-lactamase
Taq: *Thermus aquaticus*
TSA: Tryptone Soy Agar
TSB: Tryptic Soy Broth
VIM: Verona integron-encoded metallo-β-lactamase
