# Supplementary material for: Investigating the relationship between carbapenemase production and biofilm formation in Klebsiella pneumoniae clinical isolates
Source: BMC Res Notes. 2024 Feb 15;17:49. doi: 10.1186/s13104-024-06708-9 (PMC10870607; doi:10.1186/s13104-024-06708-9)
Supplement: Supplementary file 1 — Supplementary Material 1: Supplementary Figure 1. Distribution of biofilm-forming ability among carbapenemase-producing (CP-Kp) and nonproducing Klebsiella pneumoniae (CN-Kp) isolates. Supplementary Table 1.. Distribution of the biofilm-forming ability across different carbapenemase types in carbapenemase-producing Klebsiella pneumoniae (CP-Kp) isolates [file 13104_2024_6708_MOESM1_ESM.docx]

**Supplementary material**Investigating the relationship between carbapenemase production and biofilm formation in Klebsiella pneumoniae clinical isolates

**Contents:**
**Supplementary Figure 1** Distribution of biofilm-forming ability among carbapenemase-producing (CP-Kp) and nonproducing *Klebsiella pneumoniae* (CN-Kp) isolates

**Supplementary Table 1** Distribution of the biofilm-forming ability across different carbapenemase types in carbapenemase-producing *Klebsiella pneumoniae* (CP-Kp) isolates

**Supplementary Figure 1** Distribution of biofilm-forming ability among carbapenemase-producing (CP-Kp) and nonproducing *Klebsiella pneumoniae* (CN-Kp) isolates


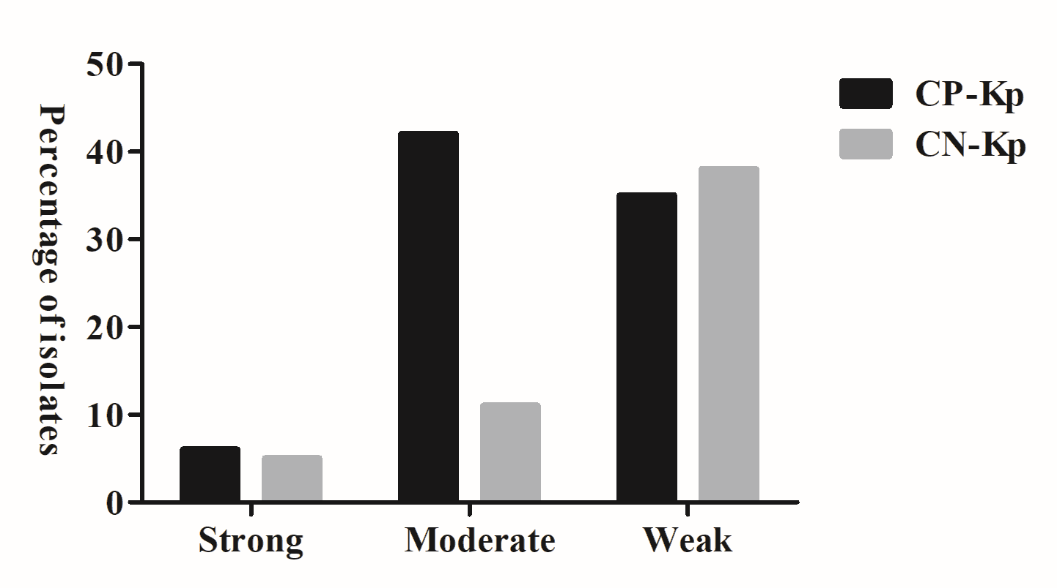


Graphical representation of the percentage of isolates in each biofilm strength category for both carbapenemase-producing (CP-Kp) and nonproducing *Klebsiella pneumoniae* (CN-Kp) isolates.

**Supplementary Table 1** Distribution of the biofilm-forming ability across different carbapenemase types in carbapenemase-producing *Klebsiella pneumoniae* (CP-Kp) isolates

| Biofilm (n) | Carbapenemase type, n (%) | | | | |
| --- | --- | --- | --- | --- | --- |
|  | NDM-1 (9) | NDM-1 and KPC (3) | | NDM-1 and OXA-48 (37) | OXA-48 (3) |
| Strong (3) | 3 (33.33) | | 0 (0) | 0 (0) | 0 (0) |
| Moderate (22) | 2 (22.22) | | 0 (0) | 18 (48.65) | 2 (66.67) |
| Weak (18) | 4 (44.45) | | 2 (66.67) | 12 (32.43) | 0 (0) |
| No biofilm (9) | 0 (0) | | 1 (33.33) | 7 (18.92%) | 1 (33.33) |

The distribution of biofilm-forming abilities among different carbapenemase types in *Klebsiella pneumoniae* isolates. For each biofilm strength category, the numbers in parentheses represent the count of isolates, and the percentages in the table cells denote the distribution of specific carbapenemase types within that biofilm category.
